# Speed-Sensitive EEG Biomarkers in a Motion Tracking Paradigm: Implications for Dynamic Visual Acuity Research

**DOI:** 10.3390/brainsci16020245

**Published:** 2026-02-22

**Authors:** Zejin Li, Guanghua Xu, Hui Li, Chenghang Du, Chengcheng Han, Xiaobing Guo, Jiahuan Wang, Sicong Zhang

**Affiliations:** 1 School of Mechanical Engineering, Xi’an Jiaotong University, Xi’an 710061, China; lizejin@stu.xjtu.edu.cn (Z.L.);; 2 State Key Laboratory for Manufacturing Systems Engineering, Xi’an Jiaotong University, Xi’an 710061, China; 3 The First Affiliated Hospital of Xi’an Jiaotong University, Xi’an 710061, China; 4 State Industry-Education Integration Center for Medical Innovations, Xi’an Jiaotong University, Xi’an 710061, China

**Keywords:** motion, target tracking, EEG feature extraction, dynamic visual acuity, V5/MT

## Abstract

**Background:** Dynamic visual acuity (DVA) is functionally distinct from static visual acuity (SVA), though SVA is often used clinically as a reference. **Methods:** To identify EEG biomarkers for DVA, we presented participants with a high-contrast checkerboard moving horizontally at speeds ranging from 4°/s to 30°/s, engaging motion-sensitive pathways while preserving spatial detail. Six EEG features—ERPs (N200 and P300), TRCA, Hjorth activity, mean curve length, and Tsallis entropy—were extracted from eight occipito-parietal channels and evaluated for speed sensitivity. **Results:** Hjorth activity and Tsallis entropy showed consistent monotonic trends with respect to speed. Hjorth activity exhibited the strongest univariate correlation (r = 0.88, *p* < 0.05). In a Lasso regression model using all speed-sensitive features, the predicted speed correlated with actual speed at r = 0.588, with TRCA-weighted features retained for their multivariate contribution. Notably, Hjorth activity peaked at PO7/PO8 (3.558 and 1.478 µV^2^ at 30°/s), aligning with V5/MT+ activation. **Conclusion:** Given its high sensitivity, neuroanatomical plausibility, and simplicity, Hjorth activity is recommended as a primary candidate for EEG-based DVA biomarker development. This study provides a foundation for objective neurophysiological evaluation of dynamic vision.

## 1. Introduction

Dynamic visual acuity (DVA) is the ability to resolve fine spatial detail in moving targets while maintaining smooth-pursuit eye movements. This capacity requires not only accurate oculomotor tracking but also efficient neural processing of motion and form, enabling observers to simultaneously follow a moving object and discriminate its features, such as the orientation of a Landolt C [1,2] gap during motion.

Current DVA assessment methods [3,4] rely heavily on behavioral measures, including eye-tracking systems and subjective participant reports (e.g., identifying the direction of a moving Snellen chart). While useful, these approaches largely overlook the underlying cortical mechanisms that support dynamic vision. While smooth-pursuit eye movements partially stabilize retinal images, their effectiveness diminishes at higher velocities (typically above 20°/s), where pursuit gain drops significantly, making neural motion-processing mechanisms critically important for DVA. Transcranial magnetic stimulation (TMS) studies [5] have demonstrated that the middle temporal area (V5/MT)—a key region for motion processing [6]—exhibits significantly stronger activation during observation of moving stimuli compared to early visual areas (V1–V3), which are primarily tuned to static features. Despite this neurophysiological evidence, few studies have leveraged electroencephalography (EEG) to characterize brain responses during DVA tasks. EEG, by contrast, offers an objective, neurophysiological window into motion-sensitive cortical processing—potentially complementing behavioral metrics with direct neural indices of dynamic visual fidelity.

Recent work by Zheng et al. [7,8] offers a promising direction: by presenting static visual targets modulated at a fixed flicker frequency, the authors successfully elicited steady-state visual evoked potentials (SSVEPs) to achieve an objective, EEG-based assessment of static visual acuity (SVA). This paradigm demonstrates that features of SSVEP can serve as an indicator of visual resolution without relying on subjective responses. However, this approach has not yet been extended to dynamic conditions, and there is currently a lack of EEG data and validated feature metrics specifically tailored to motion-based visual acuity.

To date, motion-speed sensitive EEG features remain largely unexplored. Our approach is motivated by the fact that smooth-pursuit eye movements—central to DVA—are not driven solely by retinal input but rather critically depend on cortical processing of visual information in areas V1-V5/MT. EEG thus provides a direct window into the neural substrate that links visual motion perception to oculomotor control, a core component of dynamic visual acuity that is invisible to purely behavioral measures [9]. This neuroanatomical rationale is illustrated in Figure S1 in Supplementary Material S2. This study represents the first attempt to establish an EEG-based, neurophysiologically grounded biomarker for motion-speed sensitivity in DVA. To address this gap, we propose a novel DVA stimulus paradigm in which a high-contrast checkerboard pattern flickers continuously at 7.5 Hz while moving horizontally at one of five controlled speeds (4°/s, 8°/s, 12°/s, 20°/s, and 30°/s). This design preserves the SSVEP carrier signal across both static and motion phases, ensuring that any observed neural modulation reflects changes in motion processing rather than luminance transients.

Based on prior research and pilot experiments (see the summary in Table S1 in Supplementary Material S1), we selected six EEG features for systematic comparison—encompassing time-domain measures (Hjorth activity, Hjorth mobility, and mean curve length), event-related components (ERP, N200/P300), multivariate reproducibility (task-related component analysis (TRCA)), and nonlinear dynamics (Tsallis entropy)—to capture complementary aspects of neural response under the influence of motion. These features were selected not only for their computational properties but also because they have been previously applied in studies of visually evoked cortical responses: ERP components are well-established markers of attentional engagement during motion perception [10]; Hjorth parameters have been used to quantify occipital activation during visual tracking [11]; TRCA has demonstrated success in enhancing SSVEP-based object recognition under dynamic conditions [12]; and nonlinear measures like Tsallis entropy capture complexity changes in non-stationary visual responses [13].

Subsequently, correlation analyses were conducted to examine the relationships between these features and stimulus speed and electrode locations. Accordingly, we hypothesized that among these candidate features, those most sensitive to motion-speed modulation—and exhibiting topographic specificity over V5/MT-proximal electrodes (PO7/PO8)—can serve as objective, neurophysiologically grounded biomarkers for dynamic visual acuity. The findings of this research provide a reliable reference for future evaluation methods involving multi-source signal fusion related to dynamic visual sensitivity, paving the way toward objective, neurophysiologically grounded neural correlates relevant to DVA.

## 2. Materials and Methods

### 2.1. Stimulus and Trial Design

The DVA stimuli are designed to provoke the ‘tracking’ and ‘recognition’ ability of visual processes. The ‘tracking’ ability was validated by providing the subject with a stimulus that moved at a predefined speed. The ‘recognition’ ability can be understood as SVA, which was validated by presenting the subject with a checkerboard that was converted from logMar (the logarithm of the minimum angle of resolution) based on Zheng’s study [7]. logMAR is a standardized scale where 0.0 represents decimal visual acuity (1.0), with higher values indicating poorer vision.

**Figure 1:**
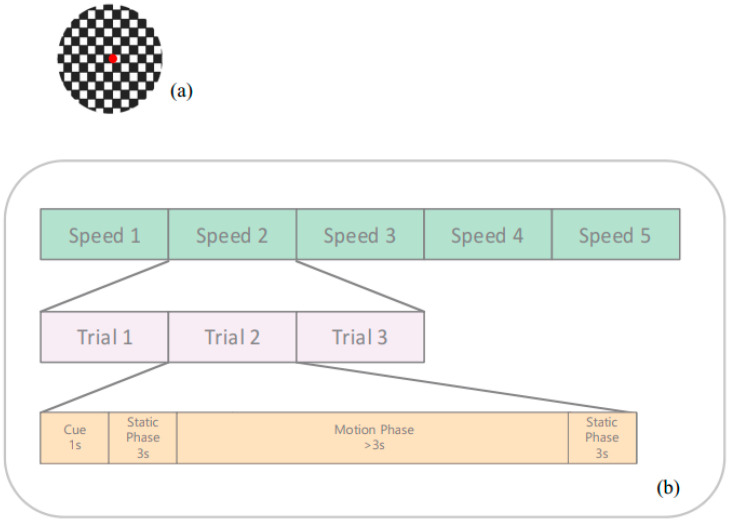
Testing procedure. (**a**) Stimulus with a red dot in the center. (**b**) Test running sequence. From top to bottom, speed 1–5 in green boxes refers to 4 deg/s, 8 deg/s, 12 deg/s, 20 deg/s, and 30 deg/s. Each speed was repeated three times, pink boxes. In each trial, see orange boxes, there were four sequential phases. In the motion phase, the stimulus moved at the speed mentioned above, one speed at a time.

In this study, we consider designing a stimulus that triggers both tracking and recognition ability. We sign the target five different moving speeds to examine the ‘tracking’ ability. According to Figure 1a, we set the checkboard resolution to match a decimal visual acuity of 0.8 (logMar = 0.1) to check ‘recognition’ ability, which is consistent with standard DVA testing protocols used in clinical and research settings [14]. Additionally, a red dot at the center of the checkboard stimulus served as a target. The white-and-black boxes in the stimulus were flipped at 7.5 Hz [15] to elicit a steady EEG response.

As shown in the bottom panel of Figure 1b, each trial comprised four sequential phases. The trials began with the presentation of a 1-s visual cue (Fix), during which a checkerboard pattern was presented at a fixed central location and flickered at 7.5 Hz. This was followed by a 3-s pre-motion static phase, in which the checkerboard continued to flicker at 7.5 Hz while remaining stationary. Next, the motion phase started: the checkerboard maintained the same 7.5 Hz contrast reversal but moved horizontally at a constant speed for a duration that varied with the speed. Finally, a post-motion static phase ensued, during which the checkerboard remained at the final position and continued flickering at 7.5 Hz for another 3 s. Throughout the entire trial, the flicker frequency, contrast, and spatial configuration of the checkerboard remained unchanged; only its spatial position was modulated during the motion phase.

### 2.2. Data Collection and Preprocessing

**Figure 2:**
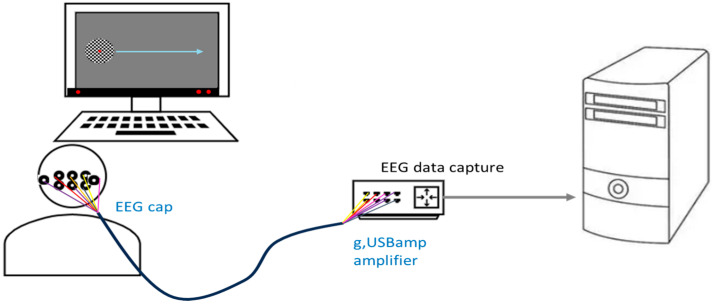
Experiment setup and data collection. The EEG amplifier (g.USBamp) record the EEG signal that were collected by EEG cap.

In this study, ten healthy participants (aged 24–32) were invited to participate in the experiment. The participants had a visual acuity of 1.0 or an acuity that was corrected (via contact lenses and glasses) to 1.0. There was no sex preference. The participants were either left- or right-handed and physically, neurologically, and psychiatrically healthy. The exclusion criteria included mental illness and abnormal vision, including due to Parkinson’s disease, alcoholism, amblyopia, strabismus, akinetopsia, etc. All the participants provided voluntary informed consent in writing after being thoroughly informed of the study’s objectives and procedures, in strict compliance with the principles outlined in the Declaration of Helsinki. Our experimental protocol was approved by the Ethics Committee of Xi’an Jiaotong University (No. 2023-1552). We created the experimental platform shown in Figure 2 to acquire data.

**Figure 3:**
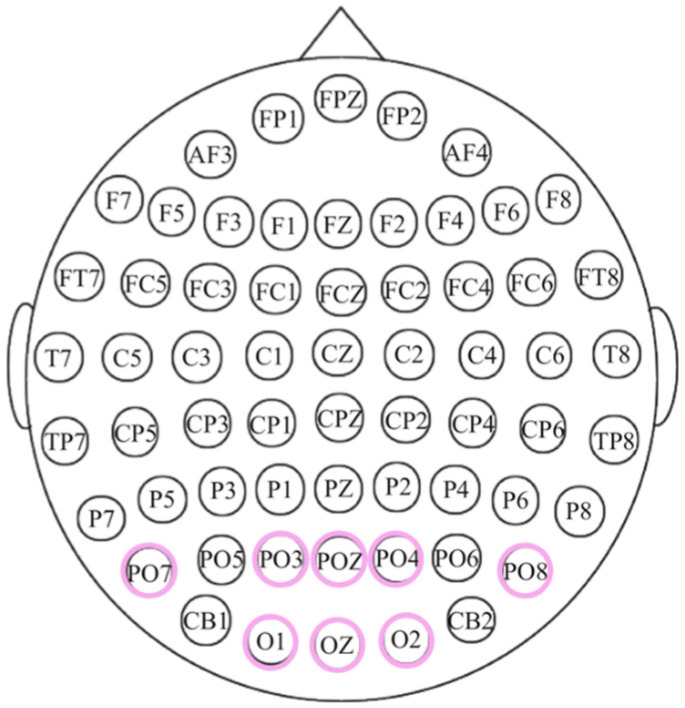
Electrode placement according to the international 10–20 system. The highlighted electrode are choosen for this research.

A g.USBamp device (manufactured by g.tec medical engineering GmbH, Schiedlberg Austria) was used to collect EEG data at a sampling rate of 1200 Hz. As shown in Figure 3, eight channels (PO3, POZ, PO4, O1, OZ, O2, PO7, and PO8) were selected. Among these, PO7 and PO8 are chosen as their proximity to the motion-sensitive areas of the occipital region [16]. Together, the EEG amplifier (g.USBamp) and active electrode system (g.GAMMAbox) formed a BCI experimental platform.

During data acquisition, hardware-based filtering was applied, for which a 48–52 Hz notch filter and a 2–100 Hz analog bandpass filter were used. Stimuli were generated using MATLAB2016b and Psychophysics Toolbox on the Windows 10 platform and displayed on an ASUS P268QN monitor with a 120 Hz refresh rate at a viewing distance of 60 cm. The observers’ heads were stabilized using a chin-and-head rest. The experiment was conducted in a room with specific lighting.

**Figure 4:**
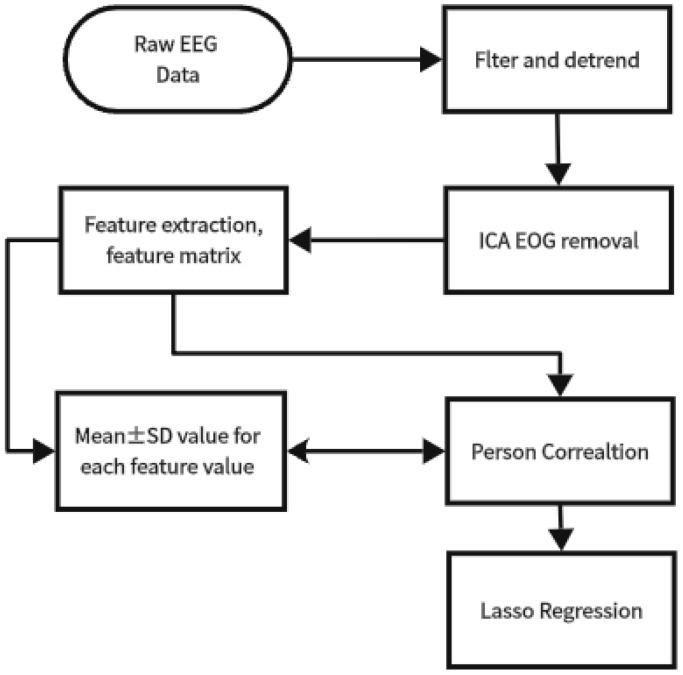
Flowchart of the data-processing procedure.

For offline analysis, further preprocessing was conducted in MATLAB using EEGLAB (v2024.0):

(1) A digital 4–40 Hz band-pass filter (zero-phase, FIR, 4th order) was applied to focus on visually evoked frequency bands;
(2) The ‘detrend’ function was used to remove linear trends;
(3) Independent component analysis (ICA) was performed using the ‘pop_runica’ function (extended Infomax algorithm) to remove ocular artifacts (blinks, saccades, etc.);

The data-processing and analysis steps are described in Figure 4.

### 2.3. Feature Extraction and Data Analysis

The representative features used to evaluate EEG signals were ERP (N200 and P300), TRCA-W, Hjorth activity, Hjorth complexity, Hjorth mobility, mean curve length (MCL), and Tsallis Entropy (see Table S1 in Supplementary Materials S1 for validation references). Below, we detail the mathematical formulations and physiological interpretations:

#### 2.3.1. Event-Related Potentials (ERPs, N200 and P300)

Let $x_{i}\left(t\right)$ denote the EEG signal in the *i*-th trial at time *t* and N denote the number of trials. As a negative-going component reflecting early visual processing, N200 is defined as the time point of minimum amplitude within the 200–300 ms window:

$$N200={\arg min}_{t\in [200,300]ms}x_{i}\left(t\right)$$

As a positive-going component associated with decision-making, P300 is defined as the time point of maximum amplitude:

$$P300={\arg max}_{t\in [300,500]ms}x_{i}\left(t\right)$$

#### 2.3.2. Hjorth Parameters

The Hjorth parameters provide critical insights into EEG signal dynamics during motion processing. There are three main Hjorth parameters. The Hjorth activity of EEG signals represents the energy or variance of the signal. The higher the level of activity, the greater the variation in the signal. The Hjorth mobility of EEG signals reflects the change in the frequency components of the signal. Higher mobility usually indicates that the signal contains more rapidly changing components. The Hjorth complexity of EEG signals pertains to the relationship between mobility and activity. Higher complexity generally suggests that the signal contains more information and more complex patterns. These parameters are defined below:

$$Activity=\sigma_{x}^{2}=\frac{1}{L}\sum_{t=1}^{L}{x(t)}^{2}$$

where *L* = total number of time points in the EEG epoch, *x(t)* = raw EEG amplitude at time *t*.

$$Mobility=\sqrt{\frac{\sigma_{x\prime }^{2}}{\sigma_{x}^{2}}},\sigma_{x\prime }^{2}=\frac{1}{L-1}\sum_{t=1}^{L-1}{\left(x\prime (t)\right)}^{2}$$

where *x’(t)* = first derivative of signal, $\sigma_{x\prime }^{2}$ = variance of the differentiated signal.

$$Complexity=\sqrt{\frac{\sigma_{x\prime \prime }^{2}\cdot \sigma_{x}^{2}}{{\left(\sigma x\prime 2\right)}^{2}}}$$

where *x″(t)* is second derivative of signal, computed as $x^{\prime \prime }\left(t\right)=x^{\prime }\left(t+1\right)-x\prime (t)\;$, $\sigma_{x\prime \prime }^{2}$ = variance of the second derivative.

#### 2.3.3. Mean Curve Length (MCL)

When applied to EEG signals, mean curve length reflects the complexity of the signal. A greater mean curve length indicates greater variability and complexity in the signal, and vice versa. Therefore, it can be used to distinguish different states of EEG activity, such as wakefulness, sleep, and epileptic seizures. Different EEG activity patterns often correspond to distinct curve shapes and levels of complexity. For multi-channel EEG data, the calculation is as follows:

$$L_{mean}^{\left(i\right)}=\frac{1}{N-1}\sum_{k=1}^{N-1}\sqrt{{\left(\frac{1}{F_{s}}\right)}^{2}}+{\left(y_{k+1}^{\left(i\right)}-y_{k}^{\left(i\right)}\right)}^{2},i=1,2,\ldots ,C$$

where *N* is the total number of sample points; $F_{s}$ is the sampling rate; *C* is the total number of EEG channels, which is 8 in this study; $y_{k}^{\left(i\right)}$ is the EEG amplitude at *k-th* sample for channel *i* and $L_{mean}^{\left(i\right)}$ is the average curve length for the $i^{th}$ channel.

#### 2.3.4. Tsallis Entropy

Tsallis Entropy quantifies non-extensive information content in EEG signals, which is critical for capturing motion-induced neural complexity. Let ${\left\{p_{i}\right\}}_{i=1}^{K}$ denote the probability distribution of the signal (e.g., from histogram bins or phase-space reconstruction) and q>0, q≠1 denote the entropic index. Then:

$$S_{q}=\frac{1}{q-1}(1-\sum_{i=1}^{K}p_{i}^{q})$$

where *q* = 2 in this study. Higher $S_{2}$ indicates greater signal randomness during dynamic movement.

#### 2.3.5. TRCA-w

Canonical correlation analysis (CCA) focuses on the spectral characteristics of the signal, whereas task-related component analysis (TRCA) effectively exploits phase information by maximizing the reproducibility of neural responses across trials within the same task condition to extract task-related components. This approach enhances inter-trial consistency while suppressing spontaneous background EEG activity.

$$w=\arg\underset{w}{\max}\frac{w^{T}Sw}{w^{T}Qw}$$

The spatial filter in TRCA is derived by solving a generalized eigenvalue problem based on the Rayleigh–Ritz theory. Specifically, given multi-trial EEG data $X_{c,tt}(t)$ for channel *C* and trial *tt*, the optimal spatial filter *w* is obtained by maximizing the ratio of inter-trial covariance to total covariance:

$$w=\arg\underset{w}{\max}\frac{w^{T}Sw}{w^{T}Qw}$$

where $S=\sum_{c=1}^{C_{task}}\sum_{tt=1}^{N_{c}}{\left(X_{c,tt}-{\bar{X}}_{c})(X_{c,tt}-{\bar{X}}_{c}\right)}^{T}$ is the inter-trial covariance matrix, Q= $\sum_{c=1}^{C_{task}}\sum_{tt=1}^{N_{c}}{X_{c,tt}X_{c,tt}}^{T}$ is the total covariance matrix, and ${\bar{X}}_{c}$ is the mean response across trials for task *C* The numerator $W^{T}SW$ amplifies reproducible motion-related components; the denominator $W^{T}QW$ suppresses spontaneous background activity.

The solution, W, is the eigenvector corresponding to the largest eigenvalue of $Q^{-1}S$. This vector W represents the relative contribution (or activation weight) of each EEG channel to the task-related component and thus serves as a meaningful neurophysiological feature. In this study, we computed TRCA using multi-trial EEG signals recorded from 8 channels, resulting in an 8 × 8 spatial filtering matrix.

The mean and standard deviation (SD) are first determined for all features at each speed and electrode. This result not only visually presents the changing trend of the features but also provides basic data for subsequent correlation analysis and regression modeling. To further explore the relationship between features and speed and brain region responses, we adopted the Pearson correlation and lasso regression measures.

The preprocessed EEG data were further extracted into features and then averaged across trials. The averaged features were organized into a 10 × 6 × 8 × 8 (subject × speed × electrode × feature) matrix based on the speed and electrodes for each subject. As shown in Figure 4, we first calculated the mean and standard deviation of each feature at each speed and electrode. The Pearson correlation and lasso regression were determined for the feature matrix and speed vector. Based on the result, a Lasso regression analysis was performed again on the speed-sensitive features. This step aimed to verify whether the speed-sensitive features also exhibit sensitivity to visual regions processing motion information.

A post hoc sensitivity analysis was conducted to determine the minimum detectable effect size for the primary relationship between the EEG feature and speed.

## 3. Results

### 3.1. Mean and Standard Deviation for Features

Feature values were trial-averaged per subject and condition. Group statistics (mean ± SD, *n* = 10) are presented in Supplementary Material Table S1. The group statistics results show that Hjorth activity and Tsallis entropy exhibit a high degree of consistency at each electrode while varying with speed, whereas the remaining features—including ERP components (N200/P300)—show neither a clear speed-dependent trend nor discernible differences across electrode sites, likely reflecting limited trial repetitions (three trials per speed) constraining ERP reliability in this rapid motion-tracking paradigm.

**Figure 5:**
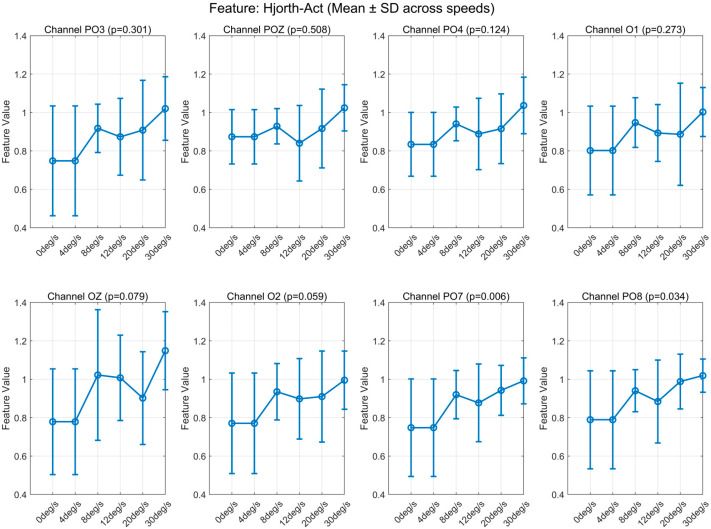
Variation in Hjorth activity with speed at each electrode.

**Figure 6:**
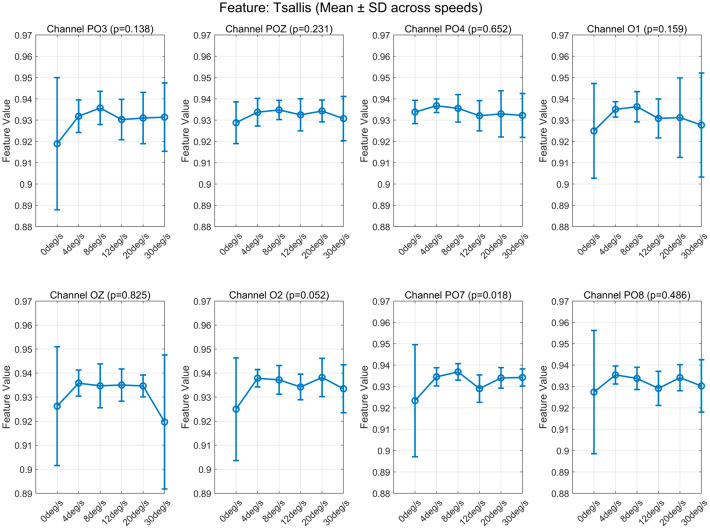
Variation in Tsallis Entropy with speed at each electrode.

We statistically analyzed the eigenvalues extracted from the eight electrodes recording EEG (see Table S2 in Supplementary Materials S1). Observation of the mean and standard deviation revealed that Hjorth activity and Tsallis entropy exhibited identical patterns of variation across all channels. Corresponding plots can be found in Figure 5 and Figure 6.

### 3.2. Validating the Correlation Between EEG Features and Speed

**Figure 7:**
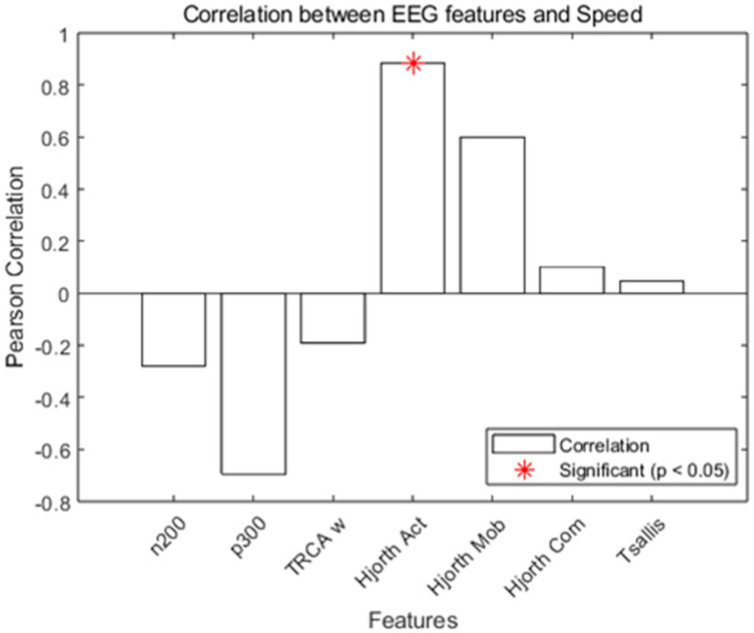
Person correlation analysis for features vs. speed.

We employed Pearson correlation and Lasso regression to evaluate the features’ sensitivity to stimulus speed. The Pearson correlation result is shown in Figure 7. Hjorth activity exhibited a strong positive correlation with speed (r = 0.88, *p* < 0.05), whereas Tsallis entropy showed no significant linear relationship, indicating insensitivity to speed variation in this framework.

**Figure 8:**
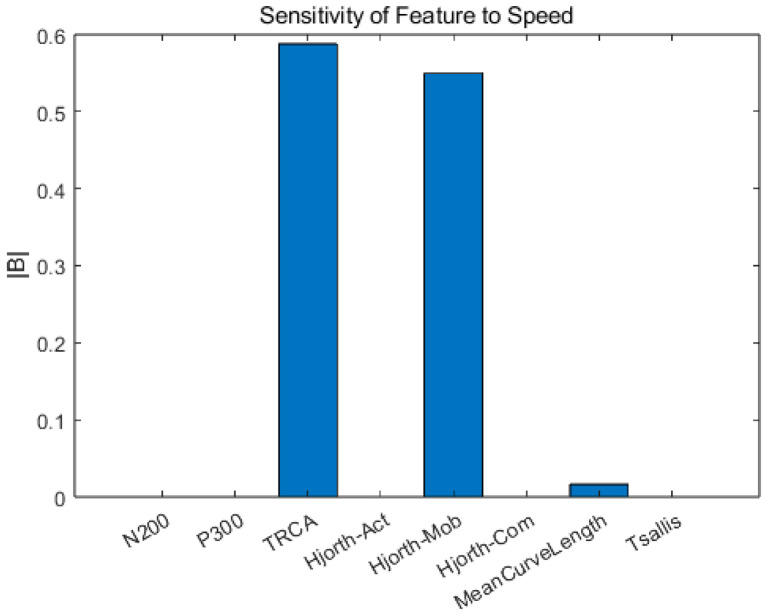
Lasso regression analysis for features vs. speed.

In the lasso regression model, TRCA-weighted (TRCA-w) features yielded the highest predictive weight (β = 0.588), followed by Hjorth mobility (β = 0.549). The results are given in Figure 8.

### 3.3. Using Lasso Regression to Verify Feature and Electrode Sensitivity

**Figure 9:**
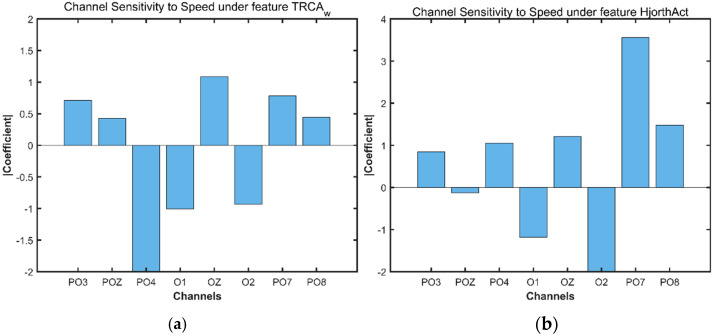
Lasso regression of the selected features’ descriptions of channel activity. (**a**) Lasso regression for TRCA-W. (**b**) Lasso regression for Hjorth activity. Lasso regression coefficients for Hjorth activity and TRCA-w at PO7/PO8 under the influence of motion (4–30°/s). Hjorth activity shows significantly higher values (PO7: 3.558 µV^2^; PO8: 1.478 µV^2^) at motion phases, consistent with V5/MT+ activation patterns.

In Section 3.2, speed sensitivity was evaluated using Pearson correlation and lasso regression, revealing that Hjorth Activity and TRCA-w are the most ideal features. In this section, Hjorth Activity and TRCA-w features are further analyzed using lasso regression to verify sensitivity to electrodes PO7/PO8, which are near the MT/V5 area of the visual cortex. PO7/PO8 are expected to be more activated during movement. Therefore, this section plots the characteristic values based on a velocity of 4–30deg/s, and static phase data is not considered. The results can be found in Figure 9, Electrodes PO7 and PO8, positioned near motion-processing visual areas (Mt/V5), demonstrated enhanced activity as expected, with Hjorth activity values of 3.558 μV^2^ and 1.478 μV^2^ (μV is the unit of EEG potential) respectively. This pattern aligns with the functional role of these regions in motion processing, confirming that the Hjorth activity metric effectively characterizes the neural variability induced by moving visual targets.

Hjorth activity exhibited a strong positive correlation with speed (r = 0.89), with 95% confidence intervals ranging from 0.72 to 0.96 across all speed conditions. See Table S3 in Supplementary Materials S1 for further details.

## 4. Discussion

Steady-state visual evoked potentials (SSVEPs) have been widely adopted in brain–computer interface (BCI) applications due to their high signal-to-noise ratios and straightforward frequency-domain extraction. Recent work has further demonstrated that SSVEP-based paradigms can serve as objective tools for assessing static visual acuity (SVA), with stimuli flickering at 7.5 Hz shown to elicit robust responses predominantly over bilateral occipital regions—centered around Oz and POz—and extending into parieto-temporal areas [8].

The human visual cortex is hierarchically organized, with early areas (V1–V3) primarily encoding static features such as shape and contrast, while the middle temporal area (V5/MT) specializes in motion perception and speed processing [17,18]. These regions interact through both feedforward and feedback pathways, and their electrophysiological responses may differ in terms of amplitude and spectral profiles depending on task demands. In our study, we observed that certain EEG features—particularly Hjorth activity and Tsallis entropy—exhibited qualitatively similar parametric trends across speeds (i.e., consistent curve shapes from 4°/s to 30°/s) despite varying absolute magnitudes across electrodes (Figure 5 and Figure 6). This suggests shared tuning mechanisms in the motion-processing network rather than uniform activation.

To specifically probe the motion-sensitive cortex, we focused on electrodes PO7 and PO8. This selection was not based on generic anatomical assumptions but rather a prior TMS mapping study [5], which found that these sites exhibit significantly stronger responses during motion observation relative to V1–V3 regions, a finding consistent with their proximity to the human MT/V5 area. While EEG data inherently lack the spatial precision of intracranial recordings and signals at PO7/PO8 may include contributions from the adjacent parieto-occipital cortex, the functional specificity of our electrode placement provides a neuroanatomically grounded basis for feature evaluation.

Our analytical pipeline reflects a two-stage, data-driven approach. First, we screened all six EEG features for speed sensitivity using Pearson correlation and lasso regression across all eight electrodes, identifying Hjorth activity and TRCA-weighted (TRCA-w) features as the top candidates. Second, we evaluated these two features for spatial specificity at PO7/PO8—the motion-sensitive sites defined by TMS. Only Hjorth activity exhibited significantly stronger modulation at these locations (Hjorth Activity value at PO7 is 3.558 µV^2^; PO8 is 1.478 µV^2^; see Figure 9b), demonstrating convergence across univariate sensitivity, multivariate retention (β = 0.549), and neuroanatomical plausibility.

We acknowledge that eye-movement artifacts can contaminate lateral occipital channels during motion tracking. To mitigate this issue, all data underwent rigorous preprocessing, including independent component analysis (ICA) with manual inspection to remove ocular components, trial rejection based on amplitude thresholds, and verification that no significant correlations existed between Hjorth activity at PO7/PO8 and EOG-like components (r < 0.1, *p* > 0.5). These steps substantially reduced the likelihood that our results reflect oculomotor artifacts.

## 5. Conclusions

DVA evaluation is crucial to clinical diagnosis [19], occupational safety, and performance [20,21,22,23], and cognitive science research [24,25]. This research provides a proof of concept for EEG-based identification of neurophysiological correlates relevant to DVA by identifying Hjorth activity as a highly sensitive biomarker for motion-speed processing. The moving stimulus paradigm—integrating motion and spatial resolution—offers a potential framework for future DVA screening tools, possibly serving as a non-invasive complement to subjective tests in certain populations, such as athletes, drivers, or patients with vestibular disorders, for whom precise motion perception is critical. Electrode localization at PO7/PO8 channels is pivotal, as their alignment with the V5/MT motion-sensitive cortical area (as validated via prior TMS mapping) underscores the need for standardized placement in future protocols to maximize signal fidelity.

However, this study’s limitations—particularly its small sample of ten healthy young adults, the absence of clinical cohorts (e.g., visually impaired individuals), and its restricted demographic diversity—require that these results be interpreted as exploratory and hypothesis-generating, rather than definitive findings. Given the small sample size and multiple feature comparisons, validation in larger cohorts with concurrent behavioral DVA measures is essential before clinical translation. Moreover, while the 7.5 Hz flicker is grounded in prior SVA research and yields stable SSVEPs over occipital regions, it represents a simplified model of natural vision; future work should compare flickering with non-flickering motion paradigms to assess ecological validity. The use of only eight EEG channels also limited spatial specificity, warranting the use of high-density arrays in follow-up studies.

## Data Availability

The data presented in this study are available on request from the corresponding author due to privacy protection and legal factors.

## Supplementary Materials

The following supporting information can be downloaded at https://www.mdpi.com/article/10.3390/brainsci16020245/s1. Tables S1–S3 can be found in supplementary material S1, Table S1: Popular EEG Features Comparison. Table S2: Mean and standard deviation of features at each electrode and speed. Table S3: Hjorth Activity result Statistic result based on speeds. Figures S1 and S2 can be found in supplementary material S2, Figure S1: Simplified oculomotor control system according to Figure 2 in Knapp’s research Knapp et al. [1]. LGN: lateral geniculate body. VN: vestibular nuclei. NOT: nucleus of the optic tract. III: cranial nerve three. VI: cranial nerve six; Figure S2 Hjorth activity values (mean ± SD across trials) for each of the 10 participants at each motion speed.
