# Supplementary material for: Speed-Sensitive EEG Biomarkers in a Motion Tracking Paradigm: Implications for Dynamic Visual Acuity Research
Source: Brain Sci. 2026 Feb 22;16(2):245. doi: 10.3390/brainsci16020245 (PMC12938763; doi:10.3390/brainsci16020245)
Supplement: Supplementary file 1 [file brainsci-16-00245-s001.zip › Supplementary Material S1.pdf]

Table S1 Popular EEG Features Comparison

Category	Feature	Advantages	Limitations	Application
Time domain	ERP[1]	High temporal resolution, directly associated with visual stimulus events (such as target recognition) data	Multiple tests need to be averaged, and the non-phase-locked components are ignored	Attention allocation and object detection in dynamic Visual acuity tasks
	Hjorth[2]	The calculation is simple and visually reflects the signal energy (Activity) and Complexity (Mobility/Complexity).	Sensitive to noise, with limited physiological explanations	Preliminarily evaluate the activation level and stability of the occipital lobe region
Frequency domain	TRCA[3]	Enhance task-related components and suppress noise	Relying on task design may result in the loss of non-task-related features	Object recognition and tracking in dynamic visual acuity tasks
Time-frequency domain	Wavelet Transfer[4]	Multi-scale time-frequency decomposition is suitable for unsteady dynamic visual signals	Parameter selection affects the result and has a high computational complexity	Analyzing frequency band energy changes during dynamic visual acuity tasks (e.g., alpha suppression)
	Phase locking value (PVL)[5]	Quantify the phase synchrony of neural oscillations and reveal the cooperative activities of visual regions	Sensitive to noise and phase estimation errors	Study the functional connections between the occipital lobe and other brain regions under visual stimulation
Nonlinear analysis	Tsallis Entropy[6]	Capturing the changes in signal complexity is suitable for dynamic adaptability research	The demand for parameter optimization is high and the physiological significance needs to be verified	Neural plasticity and information coding in the task of evaluating dynamic visual acuity
	Mean Curve Lengh (MCL)[7]	It can more comprehensively reflect the temporal dynamic characteristics of the signal without assuming the stability of the signal	Sensitive to noise, physiological significance needs to be verified	Suitable for dynamic visual tracking tasks and can quantify the neural coding complexity of the occipital lobe region for motor stimuli
	Higuchi fractal dimension (HFD)[8]	Quantify the self-similarity and complexity of the signal without assuming the signal distribution	Data length sensitive, short - time signal effect is poor	Analyze the long-term dynamic response of visual cortex to motor stimuli
Dynamic Feature analysis	Recursive covariance matrix[9]	Reveal the signal recursive characteristics and state transition, suitable for nonlinear dynamic	Computational complexity is high, the result interpretation difficult	Study the transient neural activities and mode switching in visual processing

Table S2. Mean and standard deviation of features at each electrode and speed.

Feature Name	Speed (deg/s)	PO3	POz	PO4	O1	Oz	O2	PO7	PO8
ERP-N200	0	0.07±0.23	0.04±0.18	0.02±0.18	-0.06±0.22	0.03±0.20	0.11±0.24	0.06±0.18	-0.08±0.14
	4	-0.04±0.10	0.06±0.13	0.02±0,07	-0.04±0.09	-0.03±0.04	-0.05±0.08	0.05±0.09	0.01±0.08
	8	0.00±0.11	0.06±0.07	0.03±0.09	-0.10±0.09	-0.04±0.09	-0.08±0.07	0.06±0.10	0.01±0.10
	12	0.02±0.10	0.00±0.08	0.08±0.10	-0.05±0.09	0.06±0.09	0.01±0.13	0.03±0.12	0.02±0.08
	20	-0.03±0.12	0.08±0.11	0.03±0.12	-0.06±0.09	-0.02±0.08	-0.02±0.08	-0.03±0.11	0.08±0.10
	30	0.04±0.14	0.02±0.10	0.04±0.12	-0.05±0.11	-0.05±0.13	-0.03±0.13	-0.03±0.13	0.08±0.17
ERP-P300	0	0.03±0.06	-0.02±0.10	0.00±0.08	0.02±0.09	0.00±0.07	-0.07±0.06	-0.01±0.05	0.05±0.09
	4	0.02±0.04	-0.02±0.07	-0.02±0.04	0.01±0.09	0.00±0.03	0.03±0.06	-0.02±0.08	-0.01±0.04
	8	0.00±0.10	-0.02±0.06	0.01±0.07	0.00±0.07	0.02±0.06	-0.02±0.07	-0.01±0.07	0.00±0.07
	12	0.00±0.06	0.01±0.05	0.06±0.08	-0.01±0.04	-0.02±0.04	0.00±0.08	0.02±0.07	-0.01±0.06
	20	-0.01±0.06	0.00±0.09	-0.03±0.07	0.04±0.08	-0.02±0.08	0.02±0.07	-0.02±0.07	-0.01±0.09
	30	0.01±0.07	0.03±0.11	0.01±0.08	-0.02±0.05	0.02±0.06	-0.06±0.08	0.04±0.05	-0.01±0.05
TRCA-W	0	-0.03±0.23	0.03±0.44	0.28±0.34	-0.04±0.18	0.12±0.25	0.12±0.32	-0.15±0.47	0.01±0.42
	4	0.19±0.28	0.25±0.25	0.23±0.41	0.15±0.22	0.03±0.30	0.05±0.30	-0.12±0.42	-0.03±0.42
	8	-0.03±0.39	0.08±0.50	-0.13±0.31	0.03±0.33	-0.14±0.29	0.01±0.28	0.01±0.39	-0.03±0.37
	12	0.06±0.30	0.21±0.59	0.17±0.31	0.07±0.28	0.12±0.19	-0.05±0.18	-0.12±0.38	-0.10±0.38
	20	0.02±0.44	-0.07±0.38	0.02±0.25	-0.08±0.36	0.12±0.21	-0.02±0.39	-0.06±0.45	0.12±0.35
	30	0.06±0.41	0.12±0.48	-0.01±0.31	-0.01±0.26	0.10±0.32	0.02±0.23	0.08±0.38	0.00±0.46
Hjorth-Act	0	0.75±0.29	0.87±0.14	0.83±0.17	0.80±0.23	0.78±0.28	0.77±0.26	0.75±0.25	0.79±0.26
	4	0.75±0.29	0.87±0.14	0.83±0.17	0.80±0.23	0.78±0.28	0.77±0.26	0.75±0.25	0.79±0.26
	8	0.92±0.13	0.93±0.10	0.94±0.09	0.95±0.13	1.02±0.34	093±0.15	0.92±0.13	0.94±0.11
	12	0.87±0.20	0.84±0.20	0.89±0.19	0.89±0.15	1.01±0.22	0.90±0.21	0.88±0.20	0.88±0.22
	20	0.91±0.26	0.92±0.21	0.92±0.18	0.89±0.27	0.90±0.24	0.91±0.24	0.94±0.13	0.99±0.14
	30	1.02±0.17	1.02±0.12	1.04±0.15	1.00±0.13	1.15±0.20	1.00±0.15	0.99±0.12	1.02±0.09
Hjorth-Com	0	0.83±0.03	0.78±0.08	0.83±0.04	0.80±0.04	0.80±0.08	0.81±0.05	0.84±0.03	0.80±0.08
	4	0.83±0.03	0.78±0.08	0.83±0.04	0.80±0.04	0.80±0.08	0.81±0.05	0.84±0.02	0.80±0.08
	8	0.82±0.05	0.82±0.08	0.82±0.08	0.83±0.02	0.75±0.10	0.79±0.08	0.82±0.09	0.82±0.09
	12	0.75±0.16	0.76±0.15	0.74±0.16	0.73±0.17	070±0.16	0.72±0.16	0.75±0.17	0.75±0.15
	20	0.80±0.14	0.80±0.15	0.80±0.15	0.79±0.13	0.76±0.15	0.78±0.15	0.80±0.17	0.79±0.19
	30	0.84±0.01	0.83±0.06	0.83±0.04	0.82±0.03	0.79±0.06	0.82±0.02	0.85±0.01	0.85±0.02
Hjorth-Mob	0	0.37±0.09	0.27±0.10	0.34±0.07	0.27±0.06	0.30±0.10	0.28±0.06	0.40±0.09	0.32±0.11
	4	0.37±0.09	0.27±0.10	0.34±0.07	0.27±0.06	0.30±0.10	0.28±0.06	0.40±0.09	0.32±0.11
	8	0.31±0.11	0.35±0.09	0.33±0.07	0.30±0.06	0.21±0.07	0.27±0.07	0.38±0.08	0.37±0.10
	12	0.31±0.11	0.37±0.11	0.32±0.09	0.26±0.11	0.20±0.07	0.26±0.09	0.35±0.11	0.33±0.12
	20	0.32±0.10	0.36±0.09	0.36±0.10	0.28±0.06	0.24±0.07	0.26±0.06	0.39±0.11	0.36±0.11
	30	0.034±0.07	0.38±0.10	0.34±0.11	0.29±0.08	0.25±0.08	0.27±0.05	0.40±0.07	0.39±0.07
Mean Curve Length	0	0.46±0.13	0.38±0.14	0.48±0.11	0.36±0.08	0.36±0.11	0.36±0.11	0.51±0.14	0.41±0.18
	4	0.41±0.13	0.34±0.15	0.43±0.12	0.32±0.10	0.33±0.11	0.32±0.10	0.46±0.15	0.37±0.19
	8	0.40±0.14	0.49±0.13	0.46±0.11	0.40±0.09	0.28±0.11	0.36±0.09	0.51±0.11	0.51±0.14
	12	0.38±0.18	0.45±0.18	0.40±0.15	0.31±0.15	0.25±0.11	0.31±0.16	0.43±0.19	0.42±0.18
	20	0.41±0.18	0.48±0.17	0.48±0.17	0.36±0.14	0.32±0.14	0.33±0.12	0.53±0.18	0.50±0.17
	30	0.45±0.12	0.53±0.18	0.47±0.18	0.39±0.14	0.34±0.14	0.35±0.07	0.55±0.13	0.53±0.12
Tsallis Entropy	0	0.92±0.03	0.93±0.01	0.93±0.01	0.92±0.02	0.93±0.02	0.92±0.02	0.92±0.03	0.93±0.03
	4	0.93±0.00	0.93±0.01	0.94±0.00	0.94±0.00	0.94±0.01	0.94±0.00	0.93±0.00	0.94±0.00
	8	0.94±0.01	0.93±0.00	0.93±0.01	0.94±0.01	0.93±0.01	0.94±0.01	0.94±0.00	0.93±0.01
	12	0.93±0.01	0.93±0.01	0.93±0.01	0.93±0.01	0.94±0.01	0.93±0.01	0.93±0.01	0.93±0.01
	20	0.93±0.01	0.93±0.01	0.93±0.01	0.93±0.02	0.93±0.00	0.93±0.01	0.93±0.00	0.93±0.01
	30	0.93±0.02	0.93±0.01	0.93±0.01	0.93±0.02	-0.92±0.03	0.93±0.01	0.93±0.00	0.93±0.01

Table S3 Hjorth Activity result Statistic result based on speeds

Speed (°/s)	Mean	SD	95% CI Lower	95% CI Upper	Correlation (r)
0	0.79	0.22	0.64	0.96	0.28
4	0.79	0.22	0.64	0.96	0.28
8	0.94	0.13	0.85	1.03	0.13
12	0.86	0.16	0.75	0.98	0.18
20	0.92	0.18	0.78	1.06	0.20
30	1.03	0.11	0.95	1.11	0.11

1. Hillyard, S.A.; Vogel, E.K.; Luck, S.J. Sensory Gain Control (Amplification) as a Mechanism of Selective Attention: Electrophysiological and Neuroimaging Evidence. *Philos. Trans. R. Soc. Lond., B: Biol. Sci.* **1998**, *353*, 1257–1270, doi:10.1098/rstb.1998.0281.
2. Oh, S.-H.; Lee, Y.-R.; Kim, H.-N. A Novel EEG Feature Extraction Method Using Hjorth Parameter. *Int. J. Electron. Electr. Eng.* **2014**, 106–110, doi:10.12720/ijeee.2.2.106-110.
3. Yin, X.; Liang, J.; Lin, M.; Bu, L. Task-Related Component Analysis Based on Time Filter and Similarity Constraint for SSVEP-Based BCI. *Measurement* **2024**, *235*, 114959, doi:10.1016/j.measurement.2024.114959.
4. Bajaj, N. Wavelets for EEG Analysis. In *Wavelet Theory*; Mohammady, S., Ed.; IntechOpen, 2021 ISBN 978-1-83881-947-7.
5. Bruña, R.; Maestú, F.; Pereda, E. Phase Locking Value Revisited: Teaching New Tricks to an Old Dog. *J. Neural Eng.* **2018**, *15*, 056011, doi:10.1088/1741-2552/aacfe4.
6. Capurro, A.; Diambra, L.; Lorenzo, D.; Macadar, O.; Martin, M.T.; Mostaccio, C.; Plastino, A.; Rofman, E.; Torres, M.E.; Velluti, J. Tsallis Entropy and Cortical Dynamics: The Analysis of EEG Signals. *Physica A* **1998**, *257*, 149–155, doi:10.1016/S0378-4371(98)00137-X.
7. Yahyaei, R.; Esat Özkurt, T. Mean Curve Length: An Efficient Feature for Brainwave Biometrics. *Biomedical Signal Processing and Control* **2022**, *76*, 103664, doi:10.1016/j.bspc.2022.103664.
8. Poikonen, H.; Zaluska, T.; Wang, X.; Magno, M.; Kapur, M. Nonlinear and Machine Learning Analyses on High-Density EEG Data of Math Experts and Novices. *Sci. Rep.* **2023**, *13*, 8012, doi:10.1038/s41598-023-35032-8.
9. Conte, E.; De Maio, A.; Ricci, G. Recursive Estimation of the Covariance Matrix of a Compound-Gaussian Process and Its Application to Adaptive CFAR Detection. *IEEE Trans. Signal Process.* **2002**, *50*, 1908–1915, doi:10.1109/TSP.2002.800412.
