# Supplementary material for: Speed-Sensitive EEG Biomarkers in a Motion Tracking Paradigm: Implications for Dynamic Visual Acuity Research
Source: Brain Sci. 2026 Feb 22;16(2):245. doi: 10.3390/brainsci16020245 (PMC12938763; doi:10.3390/brainsci16020245)
Supplement: Supplementary file 1 [file brainsci-16-00245-s001.zip › Supplementary Material S2.pdf]

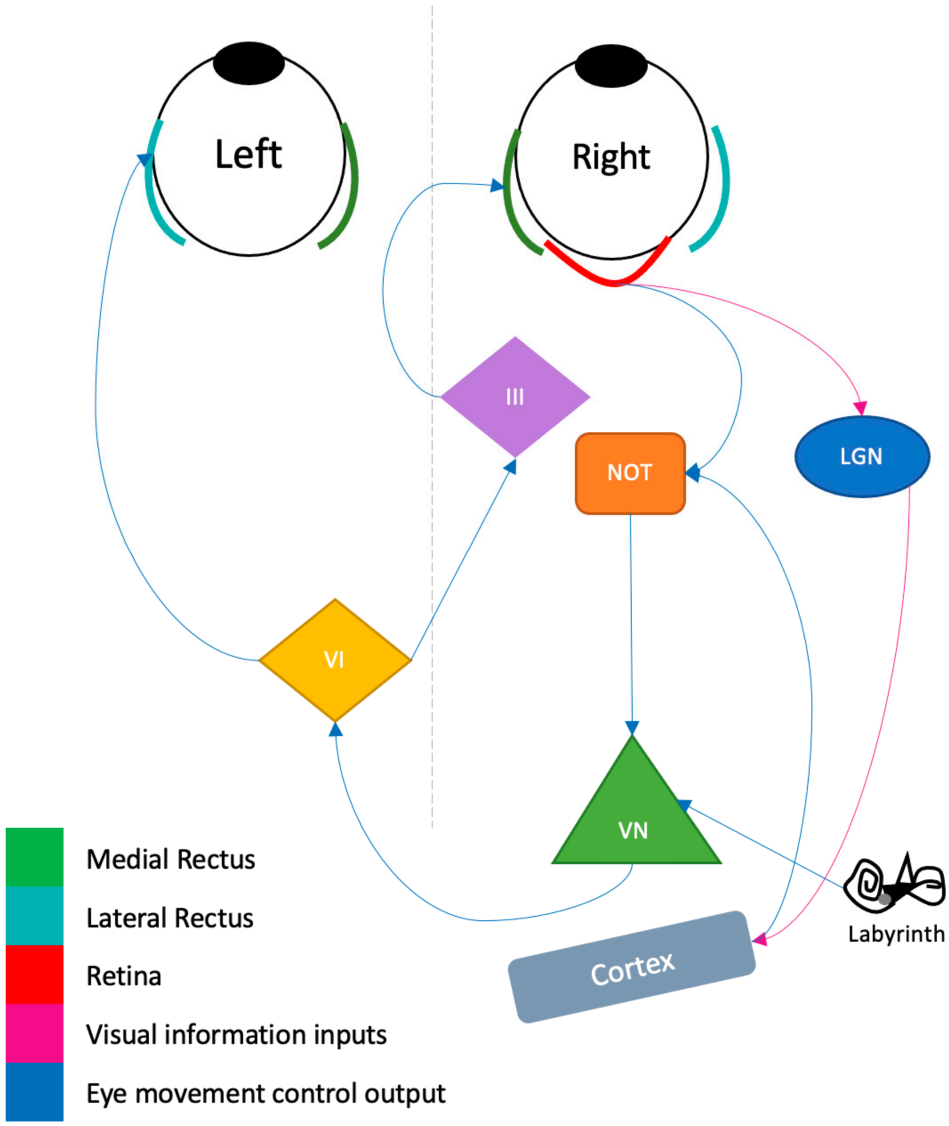

Figure S1 Simplified oculomotor control system according to Figure 2 in Knapp's research Knapp et al. [1]. LGN:lateral geniculate body. VN:vestibular nuclei. NOT:nucleus of the optic tract. III: cranial nerve three. VI: cranial nerve six.

1. Knapp, C.M.; Proudlock, F.A.; Gottlob, I. OKN Asymmetry in Human Subjects: A Literature Review. *Strabismus* **2013**, *21*, 37–49, doi:10.3109/09273972.2012.762532.

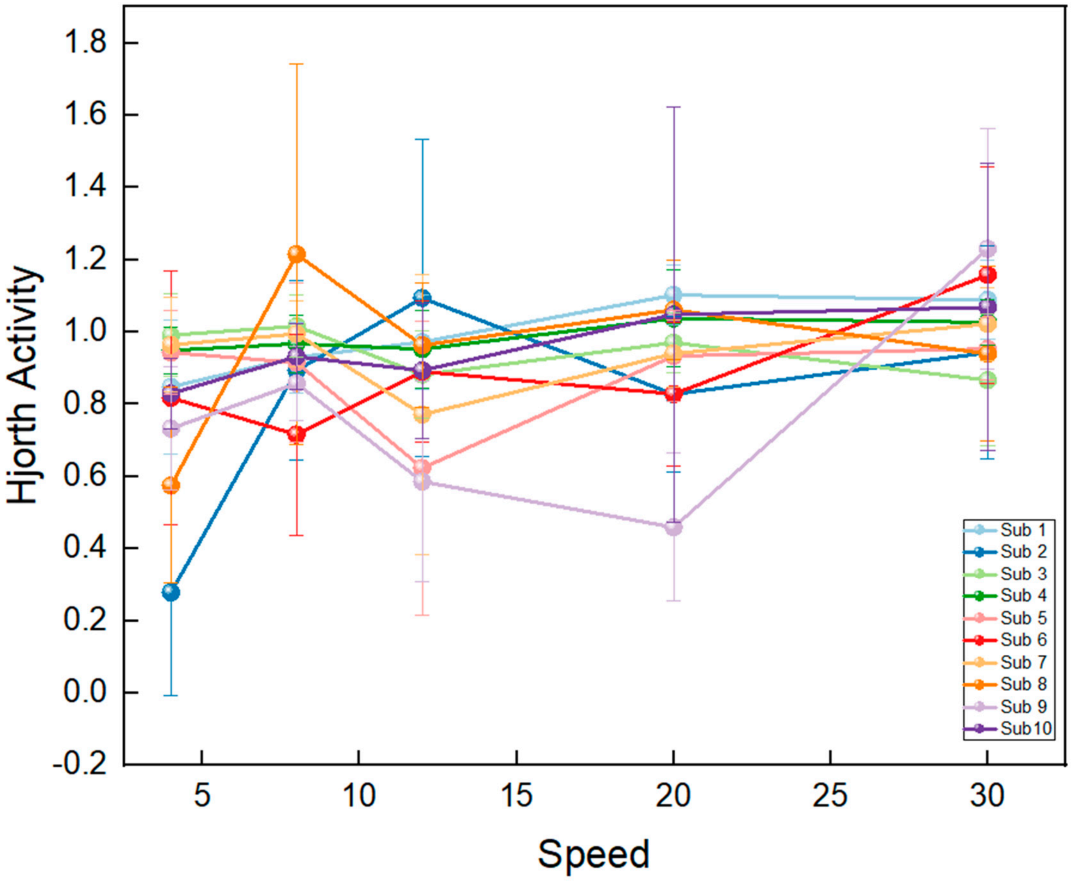

Figure S2 Hjorth activity values (mean ± SD across trials) for each of the 10 participants at each motion speed
